# Animal model integration to AutDB, a genetic database for autism

**DOI:** 10.1186/1755-8794-4-15

**Published:** 2011-01-27

**Authors:** Ajay Kumar, Rachna Wadhawan, Catherine Croft Swanwick, Ravi Kollu, Saumyendra N Basu, Sharmila Banerjee-Basu

**Affiliations:** 1 MindSpec, 8280 Greensboro Dr, Suite 150, McLean, VA 22102, USA

## Abstract

**Background:**

In the post-genomic era, multi-faceted research on complex disorders such as autism has generated diverse types of molecular information related to its pathogenesis. The rapid accumulation of putative candidate genes/loci for Autism Spectrum Disorders (ASD) and ASD-related animal models poses a major challenge for systematic analysis of their content. We previously created the Autism Database (AutDB) to provide a publicly available web portal for ongoing collection, manual annotation, and visualization of genes linked to ASD. Here, we describe the design, development, and integration of a new module within AutDB for ongoing collection and comprehensive cataloguing of ASD-related animal models.

**Description:**

As with the original AutDB, all data is extracted from published, peer-reviewed scientific literature. Animal models are annotated with a new standardized vocabulary of phenotypic terms developed by our researchers which is designed to reflect the diverse clinical manifestations of ASD. The new Animal Model module is seamlessly integrated to AutDB for dissemination of diverse information related to ASD. Animal model entries within the new module are linked to corresponding candidate genes in the original "Human Gene" module of the resource, thereby allowing for cross-modal navigation between gene models and human gene studies. Although the current release of the Animal Model module is restricted to mouse models, it was designed with an expandable framework which can easily incorporate additional species and non-genetic etiological models of autism in the future.

**Conclusions:**

Importantly, this modular ASD database provides a platform from which data mining, bioinformatics, and/or computational biology strategies may be adopted to develop predictive disease models that may offer further insights into the molecular underpinnings of this disorder. It also serves as a general model for disease-driven databases curating phenotypic characteristics of corresponding animal models.

## Background

The dramatic rise in the prevalence of autism in recent years is of major public concern [1,2]. Autism (MIM 209850) is a broad-spectrum, multifactorial condition that onsets during the first years of life with a core triad of deficits in the areas of social communication, language development, repetitive activities and restricted range of interests (DSM IV, 1994). Due to the existence of a wide range of autism-related symptoms, this complex disorder is commonly described in the context of Autism Spectrum Disorders (ASD).

A strong genetic component underlying ASD has been firmly established from various lines of studies [3-6] Genomic advances have led to the identification of hundreds of ASD candidate genes [7-11]. Recently, submicroscopic copy number variations (CNVs) were also strongly associated with ASD [9,12,13]. Furthermore, ASD is consistently associated with a number of specific genetic disorders caused by a single gene mutation, such as Fragile X Syndrome [14-17].

The high genetic heterogeneity of ASD poses an enormous challenge for understanding its etiology. For this reason, we have developed an autism gene database, AutDB, for ongoing curation of genes linked to the disorder [18]. AutDB is a disease-specific database model which curates information for all known ASD-linked genes ranging from monogenic to risk-conferring candidates. Candidate genes are richly annotated for their relevance to autism and range of molecular functions. In this manner, AutDB serves as an up-to-date, annotated resource of ASD candidate genes which provides a bioinformatics framework for understanding the pathogenesis of ASD. It is widely used by the autism research community [19-22] and is also licensed to the Simons Foundation as SFARI Gene.

In recent years, various types of animal models based on ASD candidate genes/loci linked to autism in human studies have emerged, along with the creation of numerous etiologic animal models of autism. The rapid development of ASD animal models poses a major challenge for systematic analysis of their content. Herein, we describe the design, development, and integration of an animal models database module into AutDB which comprehensively curates and catalogs ASD-related animal models. In this new "Animal Model" module, we annotate animal models with a new standardized vocabulary of phenotypic terms developed by our laboratory in order to show the correspondence of heritable traits in animal models that are relevant for the diverse clinical manifestations of ASD. This resource contains a detailed phenotypic profile for each reported animal model, presented in a user-friendly format and keyword-searchable across all tables. Each model is manually curated, cited to its references in PubMed (http://www.ncbi.nlm.nih.gov/pubmed), and cross-referenced to its entries in three external databases: Entrez Gene (http://www.ncbi.nlm.nih.gov/gene), Mouse Genome Informatics (MGI; http://www.informatics.jax.org), and Allen Brain Atlas (http://www.brain-map.org/). Moreover, entries within the Animal Model module are linked to corresponding candidate genes in the original "Human Gene" module of AutDB, thereby allowing for cross-modal navigation between gene models and human gene studies. Although the current release of the Animal Model module is limited to mouse models, it was designed with an expandable framework which can easily incorporate additional species and non-genetic etiological models of autism in the future.

Herein, we describe the design, development, and integration of a new module within AutDB, a modular, disease-specific database previously developed by this research group [18]. This work provides a platform from which data mining, bioinformatics, and/or computational biology strategies may be adopted to develop predictive disease models that may offer further insights into the molecular underpinnings of ASD.

## Construction and Content

### Data Extraction and Annotation

Content of the Animal Model module originates entirely from published, peer-reviewed scientific literature and is manually annotated by expert biologists within our laboratory. In AutDB, an "animal model" is defined as an animal in which expression of an autism-associated gene has been manipulated. A comprehensive collection of mouse models was initially compiled from a search of the scientific literature using the PubMed database at NCBI http://www.ncbi.nlm.nih.gov/pubmed with the following keywords: gene symbol and aliases, mouse/mice, and knock-out/knock-in/transgenic. Additionally, mouse models listed in review articles on autism, along with cross-references therein, were mapped and incorporated into the PubMed search lists. Once a list of animal models has been generated, it is filtered using a set of specific criteria which maintains uniformity across the entire resource: First, only mouse model reports describing observations from behavioral or neurobiological tests are selected, even if the results are negative; Second, models showing a single phenotype (i.e., embryonic lethal) without further characterization are excluded; Third, the models need to be derived from a single gene that has linked to ASD in a human genetic study. Of note, future releases will support multi-gene constructs relevant for ASD. Finally, timed daily searches ensure that the Animal Model module maintains the most up-to-date scientific content.

**Figure 1:**
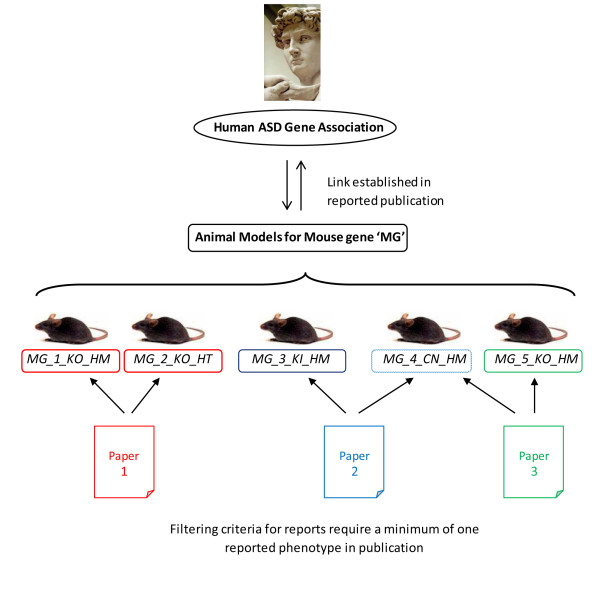
**Data Extraction for the Animal Model Database**. After a putative human candidate gene is associated to ASD and its mouse ortholog is used to create an animal model, we extract published data related to the animal model. This data consists of models curated from various reference articles. Moreover, each publication can report more than one model and/or multiple papers might report the same model, giving rise to complexity in classification.

The challenge in developing an annotation strategy for ASD animal models is to include necessary and sufficient data fields that capture various attributes of the animal model, encompass various types of constructs (knock-outs, knock-ins, knock-downs, overexpression, conditional etc.), and include the wide spectrum of phenotypes reported about ASD in the scientific literature. Once a gene has been associated with ASD, multiple publications may report animal models using differing constructs of the same gene. To add to the complexity, many publications utilize a previously reported model to extend the characterization of the phenotype. Therefore, to navigate the intricacies of the animal models, we developed a classification system that allows us to faithfully represent the reported models. Every reported model is assigned a name that consists of the gene name, chronologically ordered model number, the model construct (allele type, such as Knock Out, Knock In, etc.), and finally the genotype (Homozygous, Heterozygous, Hemizygous). We also classify publications that report the first model of a gene as "primary" with every subsequent publication recorded as "additional". This allows us to differentiate the different models reported as well as highlight models that are being repeatedly utilized. A schematic representation of the distribution of the animal model data available in the literature is shown in Figure 1.

**Figure 2:**
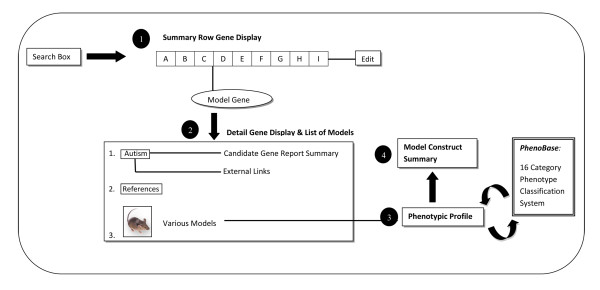
**Annotation Model for the Animal Model Database**. Animal model entries are displayed at four levels: 1) a summary row format, where each entry is annotated with gene symbol, gene name, model species, synteny, total number of model reports, total number of animal models, links to both the primary PubMed reference reporting the generation of the model and the human study for the corresponding candidate gene, and an "Edit" function; 2) a detail level showing (i) candidate gene summary with links to its entries in the external databases Entrez Gene http://www.ncbi.nlm.nih.gov/gene, Mouse Genome Informatics (MGI) http://www.informatics.jax.org, and Allen Brain Atlas http://www.brain-map.org/, (ii) references, and (iii) list of animal models; 3) the phenotypic profile of the animal model, organized under 16 categories called "PhenoBase" that are relevant for the biology of autism, and 4) a model summary providing information on the genetic construct used to create the animal model.

The Animal Model resource is constructed in a modular format with connections between sections that maximize user-friendliness (Figure 2). This system allows users to obtain complete information about the model created for each autism-specific gene. Also embedded in the database is an integrated search engine that enables users to query across the annotations based not only on the gene, but also on the observed phenotypes and model types. It is specifically designed to be expandable to accommodate additional datasets or modules as they are added. The Animal Model dataset can be searched based on gene symbol, gene name, PhenoBase category, or model type. In addition to simple searches based on a single data field, advanced queries can be built by combinations of relevant data set specific key items with restrictions on queries by way of constraints. Therefore, the combination of comprehensive data and greater data connectivity and integration provides a powerful and useful disease-based resource for biologists.

### PhenoBase

Importantly, our design of the new Animal Model module needed to address how the phenotypic profile observed in animal studies relates to the broad range of clinical manifestations of ASD reported in humans. The core behavioral domains of autism involving higher order human brain functions, such as social interaction and communications, can only be approximated in animal models. However, quantifiable and heritable traits in the mouse models can serve as markers providing mechanistic insight into the pathophysiology of the disease. Therefore, we developed an annotation model that attempts to capture and organize phenotypic data in clinically relevant domains in addition to the core behavioral features used in defining autism in humans. To this end, we developed "PhenoBase," a reference table which annotates models with new standardized phenotypic terms relevant to autism biology developed by our research team and reviewed by scientific experts on the Simons Foundation Advisory Board [23].

PhenoBase is a key component of the Animal Model module, serving as a repository of standardized phenotype terms and their definitions for annotating the animal models. To initiate the vocabulary, we first developed a high-level classification scheme encompassing 16 broad categories relevant to clinical presentations of ASD in humans (Table 1). These categories were derived from core behavioral features of ASD (social interactions & communications, repetitive behavior), together with auxiliary features of autism (seizure, mental retardation, motor phenotype, sleep pattern) observed in humans. Additionally, phenotypic characteristics reported in animal models of candidate genes (i.e., maternal behavior, an abnormality in or lack of grooming, nursing, or retrieval of pups.) were also included. Lastly, information reported on the structure and function of these genes in the brain, in animal models, was grouped under three categories of neuroanatomy/ultrastructure/cytoarchitecture, synaptic function, and molecular profile.

**Table 1:** PhenoBase.

Category ID	PhenoBase Category	Relationship to Autism	Relevance to Autism
**1**	General observations		

**2**	Social behavior	Core behavioral phenotype: Social interactions	DSM IV diagnostic criteria

**3**	Communications	Core behavioral phenotype: Communications	DSM IV diagnostic criteria

**4**	Repetitive behavior	Core behavioral phenotype: Restricted interests & Repetitive behavior	DSM IV diagnostic criteria

**5**	Maternal behavior	Broader phenotype of social memory, affect & attachment	

**6**	Motor phenotype	Auxiliary phenotype of ASD	Turner et al., 2001 [24]

**7**	Sensory	Auxiliary phenotype of ASD	Geyer and Swedlow, 1998 [25]

**8**	Learning & memory	Auxiliary phenotype of ASD	

**9**	Emotion	Hypothesis: Dysfunction of amygdala	Tsai, 1999 [26]

**10**	Seizure	25% cases	Ballaban-Gil and Tuchman, 2000 [27]

**11**	Circadian sleep/wake cycle	Auxiliary phenotype: sleep disturbances	Harvey and Kennedy, 2002 [28]

**12**	Homeostasis	Unresolved	

**13**	Inflammatory response	Auxiliary phenotype of ASD	

**14**	Synaptic function	Hypothesis: Imbalance in E/I ratio	

**15**	Neuroanatomy/Ultra- structure/Cytoarchitecture	Macrocephaly	Piven et al., 1992 [29]

**16**	Molecular profile	Unresolved	

Table depicting the 16 categories of PhenoBase, along with their relationships and relevance to autism.

To expand the scope of the Animal Model resource, each term contained within the PhenoBase was expanded to encompass both Experimental Paradigm data as well as age of testing. For each model, the appropriate terms were classified as having changed (increased, decreased, or abnormal) or not changed, depending on reports from the corresponding reference article. To maintain consistency across models, unused categories were annotated as not reported. This annotation model not only provides a complete overview of the phenotype along with pertinent supplementary information, but also allows for comparison of various types of animal models developed for each candidate gene. Additionally, this framework allows the animal models can be evaluated and scored based on the number of the ASD-related phenotype observed in the model.

To prevent overlap within PhenoBase, each category consists of multiple terms with distinct definitions based on the observations/results of a particular test. This system of classification removes focus from the individual protocol, thereby limiting overlap across categories. To illustrate this system, we depict a representative sample of the terms contained within the "Learning/Memory/Conditioning" and "Emotion" categories in Additional File 1. Although both categories contain terms with similar experimental paradigms (such as performance in different mazes), the reported results of the tests vary, warranting classification to two different categories.

Together, PhenoBase defines an integrated approach for autistic phenotypes, combining core behavioral domains of autism observed in humans with corresponding in-depth physiological and molecular characteristics reported in animal studies. The individual categories within PhenoBase are populated with terms extracted from autism-specific mouse model reports and review articles. An important consideration of our approach is to include robust terms to describe phenotypes for consistency in curation without losing their biological meaning or clinical relevance. The current version of PhenoBase includes >100 terms organized within the 16 major categories, and design of the table permits expansion of the categories with new terms and definitions as appropriate.

### Database Statistics

**Figure 3:**
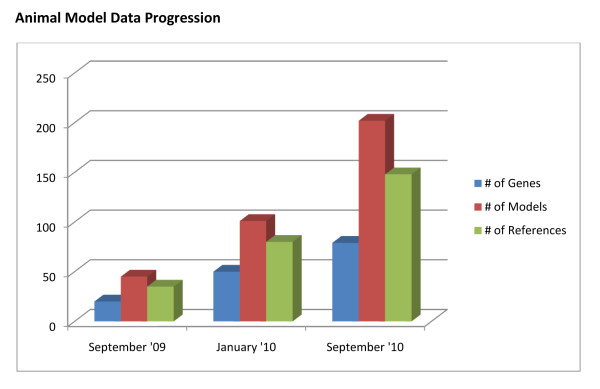
**Expansion of the Animal Model Database**. Content within the Animal Model database has rapidly accumulated over the past year. For example, the number of annotated animal models rose from 50 at the time of its beta release in September 2009 to 100 at the time of its official release in January 2010, and it now resides at >200 models. The number of corresponding genes and references within the Animal Model resource showed similar increases during this time period.

Content of the Animal Model resource has significantly expanded over the past year (Figure 3). The database was first released in September 2009, at which time it contained 50 animal models. In January 2010, the database was updated to contain a total of 100 animal models. As of September 2010, the Animal Model module encompasses >200 models, with this number still growing. The number of annotated genes and references showed similar increases during this time period so that the Animal Model module is currently based on 79 genes and almost 150 references.

**Figure 4:**
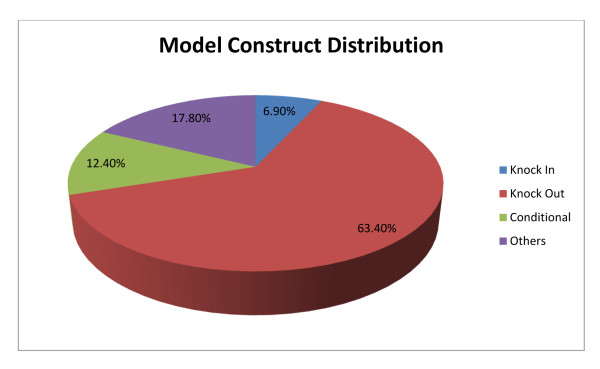
**Distribution of Genetic Models within the Animal Model Database**. With a data-freeze of September 1, 2010, we assessed the relative use of genetic constructs for creation of ASD-related animal models documented within the Animal Model database. The percentage of Knock Out models was >3 times higher than any other genetic model category.

The current distribution of genetic model types within the Animal Models module is shown in Figure 4. As of September 1, 2010, the majority of genetic constructs used to create ASD-related animal models in this resource were Knock Out (63.4%). By comparison, only a minority of animal models were created with Knock In (6.9%), Conditional (12.4%), or Other constructs (17.8%).

### Database Implementation

AutDB is a portal developed in JAVA on the J2EE platform on Linux with an RDBMS backend as its repository. AutDB is implemented in both Oracle and MySQL relational database management system where the biological information is stored. All data and images are stored in the relational database. The portal for AutDB is designed to be extensible where newer modules could be incorporated with relative ease by configuration. The application is deployed as a webapps in the Tomcat Application server connecting to the RDBMS. Connection pooling is provided by the Application Server, which greatly decreases the load on the system and enhances the performance. It also connects to the NLM database with the help of their DTDs and collects relevant information from the NLM databases for the end user. The editing, display and moderation interfaces (EDM) and automated load programs are used to display, query and input data into the AutDB system through an integrated web interface via the web browser. The EDM is an interactive, graphical interface used by scientists, curators and general users with varied role based privileges to perform their work. Thus, through EDM and automated loads, we acquire and integrate large amounts of data into a high quality, knowledgebase where the data is manually and automatically curated.

Public data access is also provided through the integrated web interface where users can interactively query and download slices of our data through a web browser.

## Utility

The Animal Model module is seamlessly integrated within the gene portal so that the data can be searched and retrieved using a single search engine. This configuration essentially links two different types of datasets: Human Gene and Animal Model. From the search page, users can select the dataset and navigate based on their requirements. The information can be searched and displayed in several ways, including complex Boolean queries. Multiple search criteria allows for individualized searches by the end user. Searching by gene name or gene symbol retrieves a gene entry that can be displayed at four levels.

The first level of display is the summary row format. As developed for the Human Gene module of the resource, each animal model report pertaining to a candidate gene was extracted, counted for the number of studies and models, and collapsed under a single header representing the model gene entry. At the summary level, each entry row is annotated with gene symbol, gene name, model species, syntenic, total number of model reports, and total number of animal models, together with a primary PubMed reference reporting the generation of the model for the candidate gene. Additionally, within the summary line display, a link to the human ASD study for the corresponding gene is provided. Moreover, the summary line display includes an "Edit" functionality that allows registered AutDB users to enter new information about an animal model. Upon approval by our research team, this new data will be incorporated into the Animal Model module.

**Figure 5:**
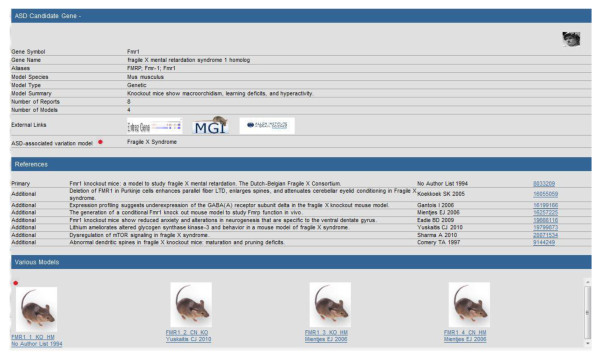
**Detail Level Display of the Animal Model Database**. At the second level of display, the new Animal Model resource provides a gene detail entry page showing (i) *ASD Candidate Gene*, summarizing the gene and providing links to its entries in the external databases Entrez Gene http://www.ncbi.nlm.nih.gov/gene, Mouse Genome Informatics (MGI) http://www.informatics.jax.org and the Allen Brain Atlas http://www.brain-map.org/, (ii) *References*, providing links to its citations in PubMed http://www.ncbi.nlm.nih.gov/pubmed, and (iii) *Various Models*, reporting the list of animal models related to that gene, where each animal model is assigned a model ID consisting of the Target Gene Symbol, chronological order number, construct type (Knock Out, Knock In, Conditional, Transgenic, Knock Down, etc.), and the genotype of the model (Homozygous, Heterozygous, Hemizygous). If the same model is reported in multiple publications, the model receives one identification number but each reference is listed and the phenotypic profile is pooled.

Each entry further displays at a detail level (Figure 5) showing: (i) *ASD Candidate Gene*, summarizing the gene and providing links to its entries in the external databases Entrez Gene http://www.ncbi.nlm.nih.gov/gene, Mouse Genome Informatics (MGI) http://www.informatics.jax.org and the Allen Brain Atlas http://www.brain-map.org/, (ii) *References*, providing links to its citations in PubMed http://www.ncbi.nlm.nih.gov/pubmed, and (iii) *Various Models*, reporting the list of animal models related to that gene.

At the third level, in-depth phenotypic characterization of the model is provided using ASD-specific annotation specifically developed for this module. The data is represented in a tabular format divided into the 16 phenotypic categories which we coined "PhenoBase" (Table 1), to be described in the next section. Each animal model with a reported phenotype is hyperlinked to its corresponding entry in PhenoBase, where any variation in phenotype of the model is color coded differently from cases of no change, thus allowing maximum comprehension for users. Alongside PhenoBase, each animal model entry is annotated with information about the experimental paradigm used and age at testing so as to allow researchers to have maximum information to conduct comparative analysis or to replicate models. Finally, the fourth level of display provides the construct definition which defines the strain of origin of mice along with the methods used to create the model.

## Discussion

Our annotation strategy involved the development of standardized terms and definitions specific to the biology of autism. The advantage of using such controlled vocabulary for defining phenotypes has been long recognized. However, a comprehensive collection of terms that is relevant for a complex human disorder with broad clinical manifestations, such as ASD, is a major challenge. With PhenoBase, we integrate core behavioral domains of autism observed in humans with corresponding in-depth physiological and molecular characteristics reported in animal studies. AutDB coupled with PhenoBase affords structured classification of a heterogeneous profile such as the phenotype data of mice. For example, multiple gene mutations that result in similar neuroanatomical changes can be filtered, thereby paving the way for elucidation of common/divergent pathways. This sort of comparison allows for consolidation of different sources of data in order to simplify data mining and analysis. By comparing results from the 16 categories of PhenoBase, researchers can more easily evaluate, score, and prioritize autism-related animal models for future ASD research.

PhenoBase is envisioned to be an ASD research community-based tool that is edited and updated by experts who are actually performing research on these mouse models. This initial draft of PhenoBase is anticipated to enable robust phenotyping of animal models and to provide a platform for further refining of the terms with precise definitions and additional attributes. For example, in the future, the models will be annotated with a series of attribute vocabulary terms that describe the quality, quantity, and character of each phenotypic term. Such refinement of PhenoBase will be accelerated by our incorporation of the "Edit" function in the summary line display of this module which allows outside researchers to provide new information about ASD animal models. In this manner, we are encouraging widespread participation from the ASD research community that will enhance AutDB as a tool for collective knowledge discovery.

The open nature of the Animal Model database implies easier expansion. In addition to adding increasing numbers of genetic models for ASD, its framework will allow us to incorporate non-genetic, etiologic models of ASD. It will also facilitate the incorporation of additional species of animal models. Moreover, the breadth of PhenoBase to encompass various terms does not limit it to a specific disease, but allows for implementation to many different disorders. For instance, Schizophrenia Gene (SZGene; http://www.szgene.org) currently includes an animal models resource but does not provide search capability similar to PhenoBase. Our development of PhenoBase permits phenotypes within the heterogeneous world of neurodevelopmental disorders to be directly compared using standardized vocabulary. With the advent of new technologies and increased identification of candidate genes for different disorders, disease-specific databases will prove to play a key role in disease biology.

With this new module of AutDB, we have created a framework that captures the phenotype of ASD animal models. This resource not only acts as a repository for disease-specific models but also allows for comparison across models. The resource has implications for the development of standards for data deposition, exchange. More importantly, it accelerates ASD research by promoting comparative analysis and prioritization of ASD animal models.

## Conclusions

Overall, we describe the design, development, and integration of a new module within AutDB for ongoing collection and comprehensive cataloguing of animal models linked to ASD. The current version of this Animal Model module curates mouse models based on manipulation of a single gene linked to ASD. Data is displayed at summary and detail levels. Moreover, the detail level of display contains a novel repository of standardized phenotype categories and terms for annotating animal models called "PhenoBase." With the integration of the new Animal Model module, AutDB provides a platform from which data mining, bioinformatics, and/or computational biology strategies may be adopted to develop predictive disease models for ASD. It also serves as a general model for disease-driven databases to systematically curate animal model phenotypes.

## Availability and Requirements

AutDB URL: http://www.mindspec.org/autdb.html

SFARI Gene URL: http://gene.sfari.org/

## Competing interests

The authors declare that they have no competing interests.

## Authors' contributions

AK performed most of the data curation and contributed to the database design. RW programmed the Java applications necessary for implementation of the database. CCS drafted the manuscript and critically analyzed the data. RK assisted in the design and implementation of the database. SNB led the design and implementation of the database. SBB guided and participated in all aspects of the project, including curation of initial database content, database design, and organizing the manuscript draft. All authors have read and approved the final manuscript.

## Authors' Information

SBB founded the nonprofit research organization MindSpec in 2006 and first released AutDB in 2007. She has over 20 years of expertise in neuroscience, bioinformatics, and systems biology.

## Pre-publication history

The pre-publication history for this paper can be accessed here:

http://www.biomedcentral.com/1755-8794/4/15/prepub

## Supplementary Material

Additional file 1 **PhenoBase Categories Avoid Overlap by Distinct Definitions of Terms**. To prevent overlap within PhenoBase, each category consists of multiple terms with distinct definitions based on the observations/results of a particular test. To illustrate this system, we depict here a representative sample of the terms contained within the "Learning/Memory/Conditioning" and "Emotion" categories. Click here for file
