# Supplementary material for: Animal model integration to AutDB, a genetic database for autism
Source: BMC Med Genomics. 2011 Jan 27;4:15. doi: 10.1186/1755-8794-4-15 (PMC3042898; doi:10.1186/1755-8794-4-15)
Supplement: Additional file 1 — PhenoBase Categories Avoid Overlap by Distinct Definitions of Terms. To prevent overlap within PhenoBase, each category consists of multiple terms with distinct definitions based on the observations/results of a particular test. To illustrate this system, we depict here a representative sample of the terms contained within the "Learning/Memory/Conditioning" and "Emotion" categories. [file 1755-8794-4-15-S1.DOCX]

| **ID**  **Additional File 1. PhenoBase Categories Avoid Overlap by Distinct Definitions of Terms** | **PhenoBase Term** | **Term Description** | **Tests** | **PhenoBase Category** | **Category Description** |
| --- | --- | --- | --- | --- | --- |
| AM8001 | *Object Recognition Memory* | anomaly in the ability to recognize objects that the animal has previously encountered; recognition is measured by relative amount of time exploring objects, which should decrease upon subsequent or multiple presentations of the same object when presented with novel objects at the same time |  | **Learning / Memory/ Conditioning** | Auxiliary phenotype of ASD: Learning & Memory Deficits |
| AM8002 | *Spatial Learning* | anomaly in the ability to ascertain or acquire spatial location information in order to improve navigation or other behavior using such location cues; spatial learning impairment in Morris water maze test | Hidden platform test; T-maze task; Barnes maze; T-maze |  |  |
| AM8003 | *Spatial Reference Memory* | anomaly in the ability to recall spatial location information from previous encounters or training sessions in order to navigate or perform other behavior using such location cues | Morris water maze task; Labyrinth maze test |  |  |
| AM8004 | *Spatial Working Memory* | anomaly in the ability to spontaneously process spatial location information in order to navigate or perform other behavior using such location cues, without previous encounters or training at that location | Delayed non-match to place task; Y-maze |  |  |
| AM8005 | *Social Transmission of food preference* | defect in the ability to recall and prefer foods associated with odors recently smelled on the breath of other mice; an indicator of defects in learning and memory of olfactory cues |  |  |  |
| AM8006 | *Cued conditioning Anomalies* | anomaly in the ability of an animal to learn and remember an association between an aversive experience (the unconditioned stimulus (US), usually a shock) and a neutral stimulus (the conditioned stimulus (CS), usually an auditory cue or light flash) | Contextual and repeated auditory cues before the conditioning electrical shocks; cued and contextual fear tasks; Adapted cued and contextual fear (CCF) assay |  |  |
| AM8007 | *Eye blink conditioning* | anomaly in the ability of an animal to learn to blink in anticipation of an aversive stimulus (e.g., an air puff to the eyelid) following repeated pairings with a neutral stimulus (e.g., a tone); such learning is only adaptive if the animal is able to learn the precise timing between the conditioned and unconditioned stimuli |  |  |  |
| AM8008 | *Cognitive flexibility* | Ability of an animal to modify previously acquired skills. | Reversal training for assessing cognitive flexibility |  |  |
| AM8009 | *Short term memory* | Ability of an animal to remember recently learned tasks. | Win-shift version of eight-arm radial maze |  |  |
| AM9001 | *Anxiety* | altered emotional response related to anticipation of a non-specific threat | Open field test; Elevated plus maze test | **Emotion** | TBD |
| AM9002 | *Response To Novelty* | amount of exploration/investigation of a novel object, situation or environment |  |  |  |
| AM9003 | *Exploratory activity* | Amount of time spent by animal exploring novel environment | Holeboard test for exploratory activity; Eight-arm radial maze test |  |  |
| AM9004 | *General curiosity* | Amount of time spent by animal exploring other animals or other objects in cage | Object preference test for general curiosity toward inanimate objects. |  |  |
| AM9005 | *Depression* | depression assayed by reduced escape attempts and/or immobility when placed in a stressful situation such as a forced swim test or a suspension test; or failure to seek pleasurable stimuli | Forced swim test; Tail suspension test |  |  |
